# The role of the environment in transmission of vancomycin-resistant *Enterococcus*: A proof-of-concept study

**DOI:** 10.1017/ash.2022.318

**Published:** 2022-11-04

**Authors:** Charlie Tan, Amber Linkenheld-Struk, Victoria Williams, Rob Kozak, Ghulam Dhabaan, Lorraine Maze dit Mieusement, Natasha Salt, Finlay Maguire, Adrienne K. Chan, Jerome A. Leis

**Affiliations:** 1 Division of Infectious Diseases, Department of Medicine, Temerty Faculty of Medicine, University of Toronto, Toronto, Ontario, Canada; 2 Sunnybrook Health Sciences Centre, Toronto, Ontario, Canada; 3 Faculty of Computer Science, Dalhousie University, Halifax, Nova Scotia, Canada; 4 Centre for Quality Improvement and Patient Safety, University of Toronto, Toronto, Ontario, Canada

Vancomycin-resistant *Enterococcus* (VRE) is an important nosocomial pathogen, accounting for ∼30% of infections by enterococci in the United States.^1^ Although asymptomatic colonization is substantially more common than infection, colonized patients are at risk of developing VRE infection, which is associated with increased morbidity, mortality, and hospital length of stay.^2^ VRE is horizontally acquired, and colonized patients, hands of healthcare workers, and the environment have been recognized as important reservoirs.^3^

We report on a unique experience prompted by the coronavirus disease 2019 (COVID-19) pandemic in our acute-care hospital that resulted in a crossover study assessing the impact of the physical environment on VRE transmission. On March 10, 2021, an outbreak of VRE was declared on a 34-bed acute-care medicine unit (ie, the outbreak unit). In total, 41 new nosocomial acquisitions of VRE were identified by April 21, 2021 (attack rate, 13%), which were confirmed as sequence type 17 (ST17) based on whole-genome sequencing (WGS). Due to the third wave of the COVID-19 pandemic in Toronto, the entire VRE outbreak cohort of (exposed) patients switched physical units with a surgical unit (nonexposed patients), to create a designated surge unit for patients with COVID-19. Both units had the same number of beds, and both were structured with a similar composition of private, semiprivate, and 3-bed ward rooms. Ultimately, neither unit was used as a dedicated COVID-19 unit. All patients with VRE colonization remained under contact precautions in private rooms with dedicated washrooms. A full description of the control measures is provided in the Supplementary Material. Prior to the unit switch, all shared areas, equipment, and patient rooms on the outbreak unit underwent terminal 2-stage cleaning with sodium hypochlorite-based solution. Following cleaning, ∼100 environmental cultures were collected, with no VRE isolated.

The unit switch proceeded on April 22, 2021, including both patients and staff, until May 18, 2021 (period 1). During this time, all patients on both units had admission, discharge, and weekly point-prevalence rectal screens for VRE. Before the unit switch was reversed, the same terminal cleaning strategies were repeated on both units. Only 1 patient from the original outbreak patient cohort remained on the non-outbreak unit before the switch back. Admission, discharge, and weekly point-prevalence screens were repeated on both units for 3 additional weeks until June 6, 2021 (period 2). To confirm that they were identical to the outbreak strain, all VRE isolates underwent WGS using the Illumina miniSeq platform (Illumina, San Diego, CA) using methods described previously.^3^ Hand hygiene adherence on both units was measured continuously across both units throughout the study using group electronic monitoring, which has been validated previously.^4^

Figure 1 summarizes the number of ST17 confirmed VRE cases and hand hygiene rates by unit during the study periods. None of the patients on the nonoutbreak unit acquired VRE during period 1 or 2, whereas 4 new acquisitions of VRE ST17 (combined attack rate, 1.1%) were observed among patients transferred to the outbreak unit (1 during period 1 and 3 during period 2). The overall hand hygiene adherence rate was 94% (141,610 of 150,706) on the outbreak unit, compared to 62% (141,589 of 227,136) on the non-outbreak unit.

**Fig. 1.**
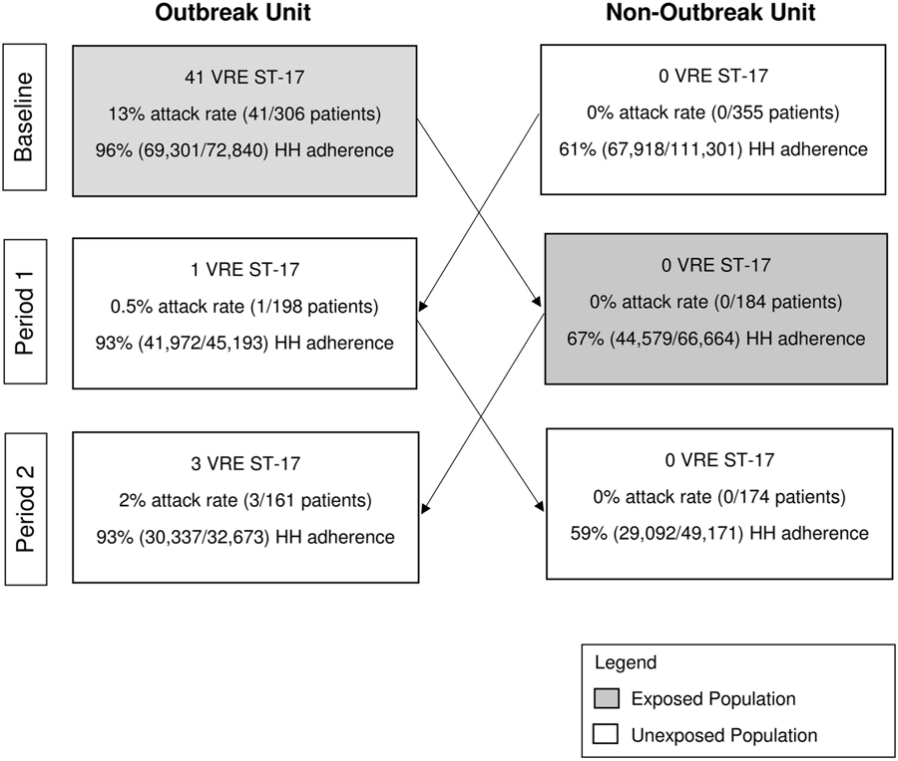
Number of vancomycin-resistant *Enterococcus* sequence type 17 (VRE ST17) acquisitions, attack rate, and hand hygiene (HH) adherence measured using group electronic monitoring system, at baseline (March 10–April 21, 2021), following crossover study period 1 (April 22–May 18, 2021) and period 2 (May 19–June 6, 2021).

Our experience serves as a proof-of-concept study regarding the role of environmental contamination in the transmission of VRE. New acquisitions occurred among both exposed and unexposed patients when located in the outbreak unit, whereas no VRE transmission occurred on the non-outbreak unit, even when there was a patient reservoir of VRE. The transmission observed, despite high hand hygiene adherence on the outbreak unit, also supports the importance of environment-to-patient transmission, independent of patient-to-patient transmission via hands of healthcare workers.

VRE is challenging to eradicate; prior studies have established the need for additional disinfection due to persistence on surfaces even after standard cleaning.^5^ A case–control study evaluating patients with newly acquired VRE found that admission to a “high-risk room,” defined as a positive environmental culture for VRE after terminal cleaning for discharge of the previous occupant, was a significant risk factor for VRE acquisition.^6^ Similarly, in a prospective crossover study of 638 critical-care admissions (50 of whom newly acquired VRE), admission to a room with prior occupation by VRE-colonized patients or with a positive environmental culture for VRE was associated with VRE transmission.^7^ Both variables remained significant predictors after controlling for antibiotic exposure and overall unit-wide burden of VRE colonization. Hayden et al^8^ showed that increases in the frequency of environmental cleaning temporally coincided with a significant reduction in VRE transmission.

VRE transmission occurred on the outbreak unit even in the absence of positive environmental cultures. Our results suggest an environmental reservoir that could not be identified and do not support environmental sampling as a routine infection control practice.^7,9^ Another consideration is unit-level antibiotic use as a driver of increased VRE rates. At our center, antibiotic use is generally higher among surgical patients than medical patients.^10^ The fact that VRE transmission occurred among the nonexposed (surgical) patients only in period 1 and not in period 2 argues against antibiotic use as a major contributor.

A strength of our study is that we performed extensive patient and environmental surveillance for VRE in both crossover periods, with WGS. We also utilized group electronic monitoring of hand hygiene, which provided an accurate picture of hand hygiene performance,^4^ though it cannot distinguish specific hand hygiene moments. Furthermore, in this observational study, VRE transmission may have been driven by other unmeasured confounding factors.

Our study reinforces the important role of environmental contamination in VRE acquisition and that environmental cleaning is a pivotal component in controlling VRE transmission.

## Supplementary material

For supplementary material accompanying this paper visit https://doi.org/10.1017/ash.2022.318. click here to view supplementary material
