# Supplementary material for: The role of the environment in transmission of vancomycin-resistant Enterococcus: A proof-of-concept study
Source: Antimicrob Steward Healthc Epidemiol. 2022 Nov 4;2(1):e178. doi: 10.1017/ash.2022.318 (PMC9641505; doi:10.1017/ash.2022.318)
Supplement: Supplementary file 1 [file S2732494X22003187sup001.docx]

**Supplemental Online Content**

| VRE Outbreak Control Measures | | |
| --- | --- | --- |
| Patient | Standard Measures | - Risk based admission screening - VRE positive patients placed in contact precautions in private rooms - Roommate exposures to VRE positive patients placed in contact precautions in private rooms, and screened on days 0, 5 and 10 post-exposure |
|  | Supplemental Outbreak Control Measures | - Universal VRE admission screening (for all admissions including internal transfers) - VRE discharge screening for all patients - Weekly VRE prevalence screen of all patients on unit - Screening of all patients transferred off unit who remain within organization (i.e., to alternate level of care) on days 0, 5, and 10 post-last exposure to outbreak unit |
| Staff | Standard Measures | - Gowns and gloves for all care for VRE positive patients |
|  | Supplemental Outbreak Control Measures | - Dedicated staff assignment to VRE positive patient cohort - Regular feedback to unit regarding new cases and performance measures (such as hand hygiene and environmental audits) |
| Environmental | Standard Measures | - Two-stage terminal clean for VRE positive patient rooms upon discharge/transfer - Cleaning and disinfection of shared equipment between patients - All cleaning performed with accelerated hydrogen peroxide-based cleaners |
|  | Supplemental Outbreak Control Measures | - Twice daily cleaning of VRE positive patient rooms - Two-stage terminal clean of all patient rooms upon discharge/transfer - Additional environmental services staff on unit for 8 hours per day - Dedicated equipment for all VRE positive patients |
| Other | Supplemental Outbreak Control Measures | - Additional environmental services staff member for cleaning of all patient equipment - Pharmacy staff member on-site for additional cleaning of medication cart - Environmental audit of unit organization and practices - Increased direct observation and practice feedback of hand hygiene (in addition to group electronic hand hygiene monitoring) |

VRE= Vancomycin Resistant Enterococcus
